# Functional restoration of conformational (R175H) and contact (R273H) mutant p53 by *Bacillus*-derived Hydroxymycotrienin A in non-small cell lung cancer: a computational approach

**DOI:** 10.3389/fbinf.2026.1875897

**Published:** 2026-07-24

**Authors:** Adeline Celina Rufus, Sidharth Kumar N, Magesh Ramasamy, Elavarashi Elangovan

**Affiliations:** 1 Medical Probiotics Lab, Department of Biotechnology, Faculty of Biomedical Sciences & Technology, Sri Ramachandra Institute of Higher Education & Research, Chennai, Tamil Nadu, India; 2 Computational Biology Lab, Department of Biotechnology, Faculty of Biomedical Sciences & Technology, Sri Ramachandra Institute of Higher Education & Research, Chennai, Tamil Nadu, India

**Keywords:** Bacillus-derived compounds, global health, Genomic stability, non-small cell lung cancer, P53 mutants

## Abstract

**Introduction:**

The TP53 mutations in Non-Small Cell Lung Cancer (NSCLC) remain a formidable clinical challenge. Current strategies, using the covalent binder APR-246, are limited by off-target toxicity and resistance.

**Methods:**

This study screens 1,580 *Bacillus*-derived metabolites to identify novel non-covalent pharmacological chaperones for native, conformational (R175H), and contact (R273H) p53 mutants. Virtual screening, ADMET profiling, molecular docking, extended 500 ns Molecular Dynamics (MD) simulations and Principal Component Analysis, against experimental anti-cancer drug APR-246, and top hit compounds.

**Results:**

Hydroxymycotrienin A emerged as the potential candidate, demonstrating thermodynamic superiority with binding affinities of −6.63 kcal/mol (R175H) and −6.57 kcal/mol (R273H), demonstrated significant superior non covalent docking affinity compared to APR-246 parent scaffold (approximately −3.5 kcal/mol) in the mutated p53 protein, suggesting a direct pharmacological chaperone activity. Unlike the covalent alkylating mechanism of APR-246, Hydroxymycotrienin A utilizes a non-covalent network to chaperone the mutant p53. MD simulations revealed that Hydroxymycotrienin A acted as a structural stabilizer for the conformational mutant R175H by suppressing atomic fluctuations within the L2 loop and reducing overall structural deviations. In the contact mutant R273H, the ligand stabilized the DNA-binding interface while maintaining favorable conformational dynamics without introducing steric clashes.

**Discussion:**

ADMET profiling predicts high bioavailability and a non-toxic safety profile, characterizing Hydroxymycotrienin A as a promising, bioavailable ‘privileged scaffold’ that offers a non-covalent therapeutic strategy for NSCLC. By restoring p53 function, this study addresses Sustainable Development Goals Target 3.4 to reduce premature mortality from non-communicable diseases. However, future *in vitro* and *in vivo* studies are required to validate the efficacy of these compounds.

## 1 Introduction

The p53 protein stands as the pivotal “guardian of the genome,” yet the TP53 gene represents the most frequently compromised barrier against human malignancy. Mapped to the short arm of chromosome 17 (17p13.1), the TP53 gene structure consists of 11 exons and 10 introns, encoding a 393 amino acid transcription factor that orchestrates a complex defense network, regulating cell cycle arrest, DNA repair, apoptosis, and cellular metabolism (Wang H. et al., 2023; Chen et al., 2022; Liu et al., 2024). In healthy physiology, p53 levels are tightly maintained at low concentrations by a negative feedback loop with the E3 ubiquitin ligase Mouse Double Minute 2 (MDM2), guiding proteasomal degradation of p53. However, under cellular stress, this interaction is disrupted, stabilizing p53 and triggering its tumor-suppressive functions (Chen et al., 2024; Klein et al., 2021). The reversal of this protective mechanism through TP53 mutations is a hallmark of oncogenesis, particularly in Non-Small Cell Lung Cancer (NSCLC), where mutations allow disease progression in nearly 60% of cases (Fan et al., 2022). These modifications differ from the enzyme-inactivating mutations characteristic of other tumor suppressors; rather than TP53, which primarily experiences missense mutations that produce stable, full-length mutant proteins with a gain of function. These are primarily classified into contact mutations (R273H), wherein residues essential for DNA binding are replaced, and structural alterations (R175H), which compromise the protein’s tertiary structure by blocking the functional DNA-binding surface (Li et al., 2020; Omar and Tuszynski, 2018). The R175H conformational mutant represents a severe structural defect; the substitution of Arginine with Histidine at position 175 disrupts the essential zinc-coordination sphere typically comprised of Cys176, His179, Cys238, and Cys242 that anchors the L2 and L3 loops (Cho et al., 1994). The loss of this ‘structural zinc’ leads to global destabilization and the functional ‘melting’ of the core domain at physiological temperatures (Bullock et al., 1997). Conversely, the R273H contact mutant maintains a native-like fold but suffers from a localized disruption of the DNA-interaction surface (Joerger and Fersht, 2008). In this variant, the loss of the positively charged Arginine side chain at residue 273 prevents the formation of critical electrostatic contacts with the DNA phosphate backbone, thereby abolishing transcriptional activity without inducing global unfolding (Cho et al., 1994).

The targeted therapy of p53 is a major challenge in contemporary oncology. For tumors with wild-type p53, strategies focus on inhibiting the MDM2-p53 interaction. However, for the high percentage of tumors harboring mutant p53, the objective is structural restoration, a task recognized as pharmacologically challenging due to the protein’s absence in profound binding cavities. The current clinical candidate, APR-246 (eprenetapopt), functions by converting to methylene quinuclidinone (MQ), which covalently binds to cysteine residues on the mutant p53 core domain, thermodynamically restabilizing it to a functional state (Hassin and Oren, 2022; Wang Z. et al., 2023). While APR-246 has demonstrated proof of concept in clinical trials, the heterogeneity of p53 mutations necessitates a broader range of therapeutic agents with distinct binding modes and reduced off-target toxicity.

Natural products serve as the cornerstone of pharmacotherapy, providing a substantial proportion of the bioactive agents currently in clinical use. The bacterial secondary metabolites have emerged as a particularly vital resource, distinguished by a structural complexity and chemical diversity that often surpasses synthetic chemical databases (Newman and Cragg, 2020; Atanasov et al., 2021). These microbial compounds possess a wide spectrum of pharmacological properties, including significant anticancer, antimicrobial, and immunomodulatory activities. Because these compounds interact with biological macromolecules, they provide “privileged scaffolds” capable of engaging tough target proteins such as p53. This evolutionary advantage allows bacterial metabolites to access binding sites and modulate protein function through mechanisms often inaccessible to standard synthetic drugs (Chatterjee et al., 2024). Historically, the investigation of these natural compounds had been hindered by prolonged isolation and screening procedures. However, the integration of high-performance *in silico* drug discovery has revolutionised this landscape. Advanced computational methods, comprising molecular docking and dynamic simulations, now enable the quick, cost-effective rational design of ligands by predicting atomic-level interactions with high fidelity (Pinzi and Rastelli, 2019).

In this study, a comprehensive *in silico* framework was used to explore the therapeutic potential of *Bacillus*-derived compounds against both wild-type and mutant (R175H and R273H) p53. By rigorously benchmarking these metabolites against APR-246, this study explores novel lead candidates that exhibit superior binding affinities and stability profiles from microbial sources. By restoring p53 function, this study directly addresses Sustainable Development Goals (SDGs) 3 (Good Health and Wellbeing) Target 3.4 to reduce premature mortality from non-communicable diseases. In this study, we focus on a ‘structural rescue strategy’ aimed at restoring the wild-type conformation to p53 mutants through the identification of novel non-covalent pharmacological chaperones.

## 2 Methods and materials

### 2.1 Ethical considerations

The study does not require ethics approval as it solely encompasses computational analysis and does not involve the use of samples from plants, humans, animals, or DNA. The study was approved by the Publications Guidelines and Monitoring Committee (PGMC) of Sri Ramachandra Institute of Higher Education and Research, Chennai, India, with the reference number SRIHER2026/PGMC134/BMS.

### 2.2 Type of sampling and reason for selection

In the study, we concentrated on examining the interplay between the target protein, human p53 coupled to the DNA-binding core domain, and its clinical drug APR-246. We explored the interactions with ligands derived from the *Bacillus* species. The choice stems from the ease with which the compounds can diffuse into the bloodstream and cells, allowing them to interact with various molecules, including proteins within the cellular environment. The comprehensive analysis served to validate our docking methodology and provided a foundational framework for comparison.

### 2.3 Inclusion criteria

The study incorporated *Bacillus*-derived metabolites from the NPatlas portal and Microbial Metabolites Database (mimeDB), whose three-dimensional (3D) structures of ligands were obtained from Pubchem.

### 2.4 Exclusion criteria

The study excluded compounds that are produced by other bacterial domains, as well as the unavailable 3D structures.

### 2.5 Protein preparation

The three-dimensional crystal structure of human p53 coupled to the DNA-binding core domain (PDB ID: 1TSR) was retrieved from the RCSB Protein Data Bank. The structure was pre-processed to remove all heteroatoms, ions, and crystallographic water molecules. Structural integrity was verified by analysing missing residues and steric clashes (Berman et al., 2000). AutoDockTools (ADT) was utilized to prepare the coordinate files by assigning Kollman charges and adding polar hydrogen atoms. The processed structure was stored as a coordinate file. For both the native and mutant models, the protein architecture, which includes loop–sheet–helix patterns typical of p53’s DNA-binding core domain, was maintained (Morris et al., 2009). Prior to mutant generation and docking studies, all crystallographic water molecules, heteroatoms, and bound ions, including the zinc ion present within the DNA-binding domain, were removed from the protein structure. Zinc removal was performed uniformly across the native p53, R175H, and R273H models to establish a consistent metal-free framework for comparative molecular docking and molecular dynamics analyses. The R175H and R273H mutant structures were subsequently generated using the mutagenesis wizard in PyMOL, followed by energy minimization to relieve local steric clashes.

### 2.6 Identification of the active pocket

The protein surface’s topological characteristics, such as solvent-accessible pockets and internal cavities, were found using the CASTp 3.0 server. Comparison with previously reported p53 ligand-binding sites revealed that the identified cavity does not overlap with the classical Cys124-containing L1/S3 pocket targeted by methylene quinuclidinone (MQ), the active metabolite of APR-246. Instead, the pocket predominantly encompasses residues within the L2 loop and neighboring structural regions, including Tyr163, Gln165, Gln167, His168, Thr170, Glu171, Val172, Arg174, Asn210, Thr211, and Phe212 (Tian et al., 2018).

### 2.7 Ligand selection

A total of 1,580 *Bacillus*-derived secondary metabolites were obtained from the Microbial Metabolites Database (mimeDB) and the NPatlas portal (Poynton et al., 2025) and subsequently cross-validated in the PubChem database (Wishart et al., 2023). Ligands were downloaded in SDF format, converted to 3D PDB format using OpenBabel, geometry-optimized, and saved as PDBQT files for docking. This comprehensive natural compound dataset allowed the identification of novel scaffolds capable of targeting mutant and native p53.

### 2.8 Pharmacokinetic analysis

The pharmacokinetic, physicochemical, drug-likeness, and toxicity profiles of the selected compounds were evaluated using ADMETlab 3.0 (Xiong et al., 2021). Canonical SMILES representations of each compound were retrieved from PubChem and used as input for *in silico* ADMET prediction analysis. Multiple parameters associated with absorption, distribution, metabolism, excretion, and toxicity (ADMET) were comprehensively assessed. Drug-likeness evaluation was performed based on Lipinski’s Rule of Five, considering criteria including molecular weight ≤500 Da, LogP ≤5, hydrogen bond donors ≤5, hydrogen bond acceptors ≤10, and acceptable physicochemical properties indicative of favorable oral bioavailability. Compounds satisfying the Lipinski criteria with minimal predicted toxicity and favorable pharmacokinetic characteristics were prioritized for subsequent molecular docking and molecular dynamics analyses. The predicted ADMET profiles and physicochemical descriptors of all screened compounds were systematically compiled and comparatively analyzed to identify potential lead candidates with enhanced drug-like properties and therapeutic suitability.

### 2.9 Virtual screening of compounds using PyRx

The crystal structure of the target protein was prepared using AutoDockTools (ADT) by adding Kollman charges and polar hydrogen atoms, followed by the assignment of Gasteiger charges. Non-polar hydrogens were merged, retaining only polar hydrogens for docking calculations. The prepared protein structure was saved in the.pdbqt format for subsequent molecular docking studies. All selected ligands underwent energy minimization prior to docking and were converted into. pdbqt format. Docking simulations were performed using the AutoDock Vina module integrated within the PyRx virtual screening platform. PyRx facilitates high-throughput virtual screening of compound libraries against specific molecular targets. An exhaustiveness parameter of eight was applied to ensure adequate conformational sampling during screening of compounds retrieved from the PubChem database. Based on AutoDock Vina binding affinity scores generated through the PyRx platform, compounds were ranked and the top 25 candidates were shortlisted for further analysis. The virtual screening step was intended solely for rapid prioritization of compounds from the large metabolite library. Subsequently, selected hits were subjected to detailed molecular docking using AutoDock 4.2.

### 2.10 Docking studies

Detailed molecular docking simulations were performed using the Lamarckian Genetic Algorithm (LGA) implemented in AutoDock 4.2 to comprehensively evaluate ligand–protein binding orientations, conformational stability, and intermolecular interaction profiles (Morris et al., 2009). Prior to docking, the receptor structure was prepared by removing crystallographic water molecules and heteroatoms, followed by the addition of polar hydrogen atoms and Kollman united atom charges, while ligand structures were energy-minimized and assigned Gasteiger partial charges and rotatable bonds to permit full torsional flexibility during docking calculations. The receptor was maintained as a rigid macromolecular framework, whereas ligands were allowed conformational freedom to extensively sample the binding landscape. Grid parameter files were generated using AutoGrid by centering the grid box around the identified active-site residues with grid dimensions of 66 × 70 × 62 points in the x-, y-, and z-axes, respectively, and a grid spacing of 0.413 Å. The grid center coordinates were defined at x = 56.973, y = 4.144, and z = 81.363 to adequately encompass the active-site cavity and surrounding interaction regions. Atom-specific affinity maps, including electrostatic and desolvation energy maps, were generated for all relevant atom types to facilitate accurate prediction of ligand binding conformations. Binding conformations were ranked based on predicted binding free energy (ΔGbinding) values derived from the AutoDock scoring function, which integrates van der Waals, hydrogen bonding, electrostatic, desolvation, and torsional energy contributions. APR-246, a well-established mutant p53 reactivator, was included as a reference control to validate the docking protocol and facilitate comparative assessment of binding affinities, interaction stability, and active-site occupancy. Post-docking interaction analyses and structural visualizations were performed using BIOVIA Discovery Studio to characterize hydrogen bonds, hydrophobic interactions, π–π stacking, π–alkyl interactions, electrostatic contacts, and other non-covalent interactions contributing to ligand stabilization within the protein binding pocket.

### 2.11 Molecular dynamics simulations (MDS)

Molecular dynamics simulations (MDS) of the protein–control and protein–test ligand complexes were performed using GROMACS 2025.1 to investigate the conformational stability, structural compactness, and dynamic interaction behavior of the ligand-bound target protein systems. The AMBER force field was employed for protein parameterization, while ligand topologies were generated using the Antechamber module of AmberTools with the General AMBER Force Field (GAFF2). Partial atomic charges were assigned using the AM1-BCC charge model prior to topology generation and subsequent molecular dynamics simulations. The prepared protein–ligand complexes were positioned at the center of a cubic simulation box and solvated using explicit water molecules, followed by the addition of suitable counter-ions to neutralize the net system charge. Energy minimization was subsequently carried out using the steepest descent algorithm to eliminate unfavorable steric contacts and obtain energetically stable conformations prior to equilibration. System equilibration was achieved under canonical (NVT) and isothermal–isobaric (NPT) ensembles to stabilize temperature and pressure conditions throughout the simulation environment. After equilibration at 300 K and 1 bar pressure, a 500 ns production MD simulation was performed. Post-simulation trajectory analyses, including Root Mean Square Deviation (RMSD), Root Mean Square Fluctuation (RMSF), Radius of Gyration (Rg), Hydrogen Bond (H-bond), and Solvent Accessible Surface Area (SASA), were conducted to evaluate structural stability, flexibility, intermolecular interactions, and compactness of the complexes throughout the simulation period (Priya et al., 2025).

To further investigate the large-scale collective motions and conformational dynamics of the protein–ligand complexes during the simulation trajectory, Principal Component Analysis (PCA) was performed using covariance-based essential dynamics analysis (Priyanka et al., 2025). PCA decomposes atomic motion into a series of orthogonal eigenvectors and corresponding eigenvalues derived from the covariance matrix of backbone atomic fluctuations, thereby enabling identification of dominant conformational transitions and correlated motions governing protein dynamics. The covariance matrix was generated using the gmx covar module of GROMACS 2025.1 based on backbone atom coordinates, and the resulting eigenvectors and eigenvalues were analyzed using the gmx anaeig module. Projections of the MD trajectories along the principal components were subsequently examined to characterize conformational sampling and motion distribution within the protein structure.

### 2.12 Free Energy Landscape (FEL)

Free energy landscape analysis was performed to characterize the conformational stability and dynamics of the protein–ligand complexes (Amadei et al., 1993). Following MD simulation, the trajectories were first corrected for periodic boundary conditions and least-squares fitted to the initial reference structure to remove global translational and rotational motions. Principal component analysis (PCA) was then carried out using the Cα atoms to construct the covariance matrix of atomic fluctuations, which was diagonalized to obtain eigenvectors and eigenvalues representing the dominant collective motions. The first two principal components (PC1 and PC2), accounting for the largest variance in the system, were selected to describe the conformational space. The free energy landscape was calculated by projecting the MD trajectories onto the PC1–PC2 plane using the Boltzmann relation G = −kBTln-PG = -k_BT \ln PG = −kB​TlnP, where kBk_BkB​ is the Boltzmann constant, TTT is the simulation temperature, and PPP denotes the probability distribution of sampled conformations (Maisuradze et al., 2010; Muthukumar et al., 2026). Two-dimensional contour maps and three-dimensional surface plots were generated to visualize the distribution, depth, and connectivity of low-energy basins, enabling comparative assessment of conformational stability and flexibility across the different protein–ligand systems.

## 3 Results

### 3.1 Target protein data collection and identification of the binding site

The three-dimensional structure of the human p53 DNA-binding core domain was successfully retrieved and processed for computational analyses. CASTp evaluation revealed a prominent surface pocket positioned within the DNA-binding interface, characterized by an interconnected cavity system extending across residues Ser96, Thr170, Val97, Asn210, Phe212, Thr211, and adjacent loop regions. This pocket demonstrated sufficient depth, volume, and solvent accessibility to accommodate small-molecule ligands. Structural inspection of the mutant models showed that the R175H mutation caused noticeable distortion within the L2 loop; in contrast, the residue directly associated with DNA interaction was compromised by the R273H mutation. Despite these alterations, both mutant structures retained the major CASTp-identified pocket, allowing all subsequent docking and simulation analyses to be conducted uniformly across the native and mutant p53 variants.

### 3.2 Ligand retrieval

A comprehensive library comprising 1,580 *Bacillus*-derived secondary metabolites was systematically curated using two authorized natural product repositories: the Microbial Metabolite Database (mimeDB) and Natural Product Atlas (NPAtlas). These databases were selected due to their extensive annotation of microbial natural products, including experimentally validated compounds and structurally characterized metabolites reported in the literature. All relevant *Bacillus*-associated metabolites were filtered based on organism source, metabolite classification, and availability of structural information. The chemical structures of the selected metabolites were downloaded in Structure Data File (SDF) format to preserve complete molecular information, including atomic coordinates, bond connectivity, stereochemistry, and physicochemical annotations. Subsequently, the compounds were subjected to a cross-validation process against the PubChem database to ensure structural accuracy, eliminate redundancy, and confirm the chemical identity. This validation involved verification of compound identifiers (CID), molecular formula, canonical SMILES, InChI, and structural consistency. Duplicate entries and incomplete structures were carefully removed, and discrepancies were resolved by referencing the PubChem canonical structure.

### 3.3 Virtual screening of the retrieved ligand molecules

A total of 1,580 *Bacillus*-derived secondary metabolites were screened against the p53 active pocket using PyRx. Virtual screening produced a clear stratification of ligands based on predicted binding affinity, enabling the selection of the highest-scoring molecules for further evaluation. Among these, seven metabolites emerged with particularly favorable binding energies, that is enlisted in Table 1, including Hydroxymycotrienin A, a chrysen-derived polycyclic compound, Macrolactin I, Onoceranoxide, Cereusitin A, Sporulene B, and Hydroxymycotrienin B. Hydroxymycotrienin A consistently ranked as the top candidate, displaying the strongest predicted affinity in comparison with the remaining compounds. The superior geometric fit of Hydroxymycotrienin A can be attributed to its triene-ansamycin architecture. The 22-membered macrocyclic ‘ansa’ bridge allows the molecule to adopt a semi-rigid, crescent-shaped conformation that effectively binds the shallow DNA-binding pocket of p53. Furthermore, the conjugated triene system provides a planar hydrophobic surface that facilitates robust interactions, specifically the Pi-Alkyl and Pi-Sigma contacts with His168 and Phe212. The presence of specific hydroxyl groups at the macrocyclic periphery further enables the formation of critical, directional hydrogen bonds with Thr170, a residue essential for maintaining the stability of the L2 loop.Subsequent ADME and toxicity filtering retained the most pharmacologically favorable candidates, narrowing the pool to two major ligands: Hydroxymycotrienin A and Macrolactin I. While candidates such as Hydroxymycotrienin B demonstrated high affinity, they were designated as ‘FAIL’ in the ADMETlab column due to high predicted probabilities (>0.70) of hERG inhibition and hepatotoxicity in the consensus models. Hydroxymycotrienin A demonstrated superior drug-likeness, an acceptable toxicity class, and physicochemical properties conducive to structural binding. Detailed chemical identifiers, including canonical SMILES strings and InChIKeys for these compounds, are provided in Supplementary Table S1 to facilitate reproducibility and further structural studies.

**TABLE 1:** Virtual screening and predicted AMET profiles of the top seven hits of *Bacillus*-derived metabolites against p53 protein.

Pubchem ID	Name	Binding affinity (kcal/mol)	ADMETlab 3.0	Protox
139,587,614	Hydroxymycotrienin A	−8.1	PASS	6
139,583,555	(1R,2R,4aS,4bR,6aS,10aS,12aS)-2.4b,7,7,10a,12a-hexamethyl-1-[3-(4-methylphenyl)butyl]-3,4,4a,5,6,6a,8,9,10,10b,11,12-dodecahydro-1H-chrysen-2-ol	−7.7	PASS	5
6,390,938	Macrolactin I	−7.6	PASS	5
21,672,657	Onoceranoxide	−7.5	PASS	5
132,494,419	Cereusitin A	−7.3	PASS	5
102,596,711	Sporulene B	−7.2	PASS	6
139,583,958	Hydroxymycotrienin B	−7.2	FAIL	6

Binding affinities presented in Table 1 were obtained from the AutoDock Vina scoring function within the PyRx virtual screening workflow and were used exclusively for compound ranking. Following virtual screening, the top candidates will be subjected to detailed docking using AutoDock 4.2.

### 3.4 ADME and toxicity analysis

Pharmacokinetic evaluation showed that most top-screened ligands fulfilled essential drug-likeness criteria, including Lipinski compliance and favorable solubility profiles. Hydroxymycotrienin A exhibited optimal molecular weight, logP values, hydrogen bonding capability, and a high predicted gastrointestinal absorption score. Toxicity profiling through ProTox-II categorized the compound within a low-risk class, indicating minimal systemic toxicity. Macrolactin I also displayed acceptable ADME features but demonstrated slightly higher predicted toxicity and reduced bioavailability compared with Hydroxymycotrienin A. Based on these cumulative assessments, Hydroxymycotrienin A was advanced as the principal ligand for detailed docking and molecular dynamics simulations.

### 3.5 Molecular docking of p53 protein

**FIGURE 1:**
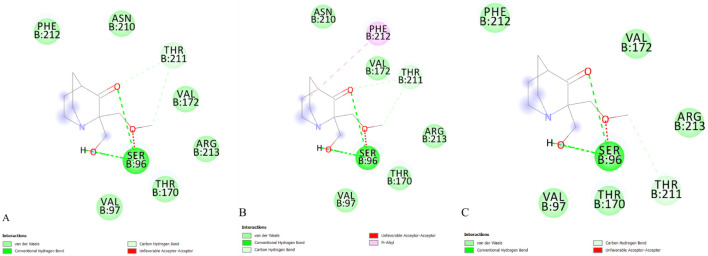
The 2D molecular docking interaction of the native p53 and both the mutated p53 proteins (R175H and R273H) with APR-246.

AutoDock-based molecular docking revealed that Hydroxymycotrienin A formed stable and energetically favorable interactions with native p53, exhibiting a binding affinity of −5.87 kcal/mol, which surpassed the affinity of APR-246, the experimental anti-cancer molecule, which bound with an affinity of −3.51 kcal/mol. The ligand established a network of interactions involving Thr170 through hydrogen bonding, van der Waals contacts with residues such as Gln167, Glu171, Val172, Phe212, Asn210, and Ser96, and hydrophobic stabilization through Pi-alkyl interactions with His168, which is depicted in Figure 1. These interactions positioned the ligand deeply within the CASTp-defined cavity, suggesting potential to reinforce structural stability.

**FIGURE 2:**
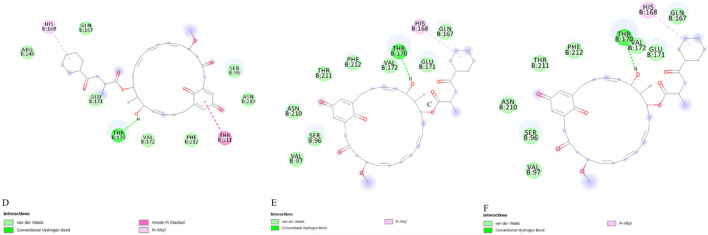
The 2D molecular docking interaction of the native p53 and both the mutated p53 proteins (R175H and R273H) with Hydroxymycotrienin A.

Docking results for the R175H mutant indicated a further improvement in binding affinity, with Hydroxymycotrienin A binding at −6.63 kcal/mol, nearly twice as favorable as APR-246, which showed a docking score of −3.56 kcal/mol. The ligand engaged with key residues including Thr170, Val97, Ser96/97, Asn210, Thr211, Phe212, Val172, and acidic residues situated around the L2 loop, as pictured in Figure 2. The retention of Pi-alkyl interactions with His168 and the presence of stabilizing hydrogen bonds suggested a compensatory effect on mutation-induced structural disruption.

**FIGURE 3:**
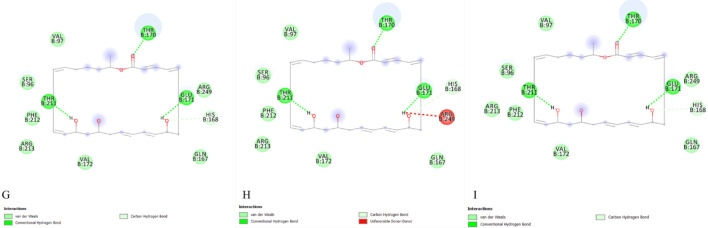
The 2D molecular docking interaction of the native p53 and both the mutated p53 proteins (R175H and R273H) with Macrolactin I.

Similarly, docking with the R273H mutant demonstrated strong ligand accommodation, with Hydroxymycotrienin A binding at −6.57 kcal/mol. Interactions in this complex included conventional hydrogen bonding with Thr170 and a wide array of van der Waals contacts spanning conserved residues crucial for DNA binding, which is illustrated in Figures 2, 3. APR-246 again demonstrated a weaker affinity at −3.52 kcal/mol. These results collectively indicated that Hydroxymycotrienin A maintained consistent structural engagement across native and mutant p53 forms, outperforming the benchmark molecule in all three docking scenarios. The overall docking scores, residues, and bonds for native p53 and both the mutations are presented in Table 2.

**TABLE 2:** Molecular docking scores obtained from auto dock software for the native p53, R175H mutation, and R273H mutation against APR 246 and Hydroxymycotrienin A.

Proteins	Ligand/Drugs	Docking score (kcal/mol)	Residues	Type of bonds
Native p53	APR-246	−3.51	Ser 96	Conventional hydrogen bond
			Phe 212, Asn 210, Val 172, Arg 213, thr 170, Val 97	Vanderwaal interaction
			Thr 211	Carbon hydrogen bond
	Hydroxymycotrienin A	−5.87	Thr 170	Conventional hydrogen bond
			Gln 167, Arg 249, glu 171, Val 172, phe 212, Asn 210, ser 96	Vanderwaal interaction
			Thr 211	Amide-pi interaction
			His 168	Pi-alkyl
Mutation R175H	APR-246	−3.56	Ser 96	Conventional hydrogen bond
			Asn 210, Val 172, Arg 213, thr 170, Val 97	Vanderwaal interaction
			Thr 211	Carbon hydrogen bond
			Phe 212	Pi-alkyl
	Hydroxymycotrienin A	−6.63	Thr 170	Conventional hydrogen bond
			Val 97, ser 97, Asn 210, thr 211, phe 212, Val 172, glu 171, glu 167	Vanderwaal interaction
			His 168	Pi-alkyl
Mutation R273H	APR-246	−3.52	Ser 96	Conventional hydrogen bond
			Phe 212, Val 172, Arg 213, thr 170, Val 97	Vanderwaal interaction
			Thr 211	Carbon hydrogen bond
	Hydroxymycotrienin A	−6.57	Thr 170	Conventional hydrogen bond
			Val 97, ser 96, Asn 210, thr 211, phe 212, Val 172, glu 171, gln 167	Vanderwaal interaction
			His 168	Pi-alkyl

### 3.6 Molecular dynamics

**FIGURE 4:**
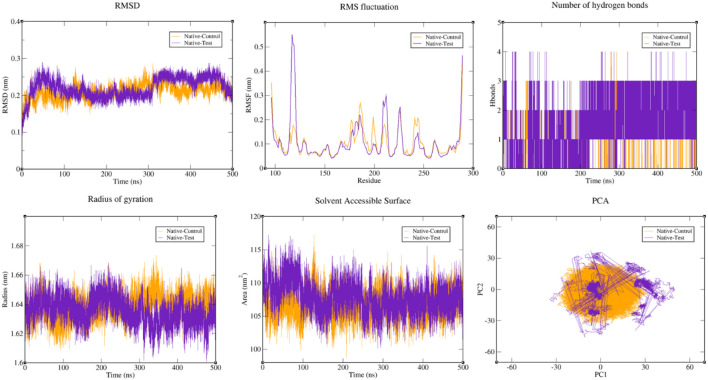
Molecular dynamics simulation analysis (500 ns) evaluating the structural stability of the native p53 DNA-binding domain upon binding with Hydroxymycotrienin A.

The 500 ns molecular dynamics trajectories demonstrated distinct differences in conformational stability, residue flexibility, compactness, and collective motion between the APR-246 control systems and the Hydroxymycotrienin A-bound complexes across native and mutant p53 structures. In the native p53 system, the Hydroxymycotrienin A complex rapidly attained equilibration within the initial simulation phase and maintained a comparatively narrow RMSD distribution throughout the trajectory, indicating preservation of backbone structural integrity under prolonged dynamic conditions (Figure 4A). In contrast, the APR-246-bound control exhibited persistent conformational drift and larger backbone deviations, reflecting reduced structural convergence. RMSF analysis further revealed suppression of residue-level fluctuations in the ligand-bound system, particularly within the DNA-binding interface and loop regions surrounding residues 165–215, suggesting that Hydroxymycotrienin A dampened local flexibility within functionally important structural elements (Figure 4B). The hydrogen bond profile demonstrated sustained intermolecular interactions with minimal fluctuation frequency over the simulation period, supporting prolonged ligand retention within the binding cavity (Figure 4C). Consistently, Radius of Gyration (Rg) analysis showed maintenance of a compact folded architecture with reduced structural expansion relative to the control complex (Figure 4D), while SASA calculations indicated decreased solvent exposure of the protein surface following ligand accommodation (Figure 4E). Principal Component Analysis (PCA) demonstrated that the Hydroxymycotrienin A-bound system occupied a highly confined conformational phase-space characterized by dense clustering along PC1 and PC2 coordinates, whereas the APR-246 complex sampled broader conformational distributions indicative of increased global mobility and reduced dynamic convergence (Figure 4F).

**FIGURE 5:**
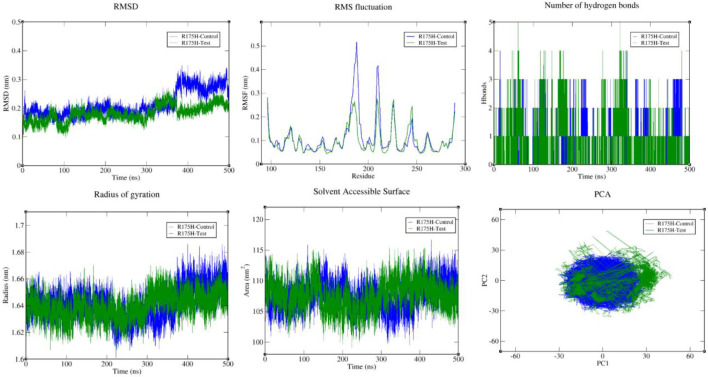
Structural remediation of the R175H p53 conformational mutant through Hydroxymycotrienin A binding during 500 ns molecular dynamics simulations.

The R175H conformational mutant exhibited pronounced structural destabilization in the APR-246 control trajectory, characterized by elevated RMSD oscillations and delayed equilibration over the simulation timescale (Figure 5A). Upon binding with Hydroxymycotrienin A, the mutant complex demonstrated marked attenuation of backbone deviations and significantly improved trajectory convergence, indicating effective stabilization of the mutation-disrupted structural framework. RMSF analysis revealed a substantial reduction in atomic fluctuations within the L2 loop region and adjacent DNA-binding residues, highlighting the ability of the ligand to suppress local conformational mobility associated with the R175H mutation (Figure 5B). Persistent intermolecular hydrogen bonding throughout the trajectory further supported stable ligand occupancy and interaction persistence within the active pocket (Figure 5C). The Rg profile demonstrated reduced conformational expansion and preservation of globular compactness in the test complex compared with the more fluctuating control system (Figure 5D), while SASA analysis suggested partial burial of exposed surface regions following ligand stabilization (Figure 5E). PCA projections showed that the Hydroxymycotrienin A-bound R175H complex sampled a restricted low-energy conformational basin with limited phase-space dispersion, whereas the control mutant explored multiple metastable conformations associated with structural instability (Figure 5F). Collectively, these findings indicate that Hydroxymycotrienin A functions as a structural stabilizer capable of suppressing mutation-induced conformational disorder and enhancing the dynamic stability of the R175H mutant.

**FIGURE 6:**
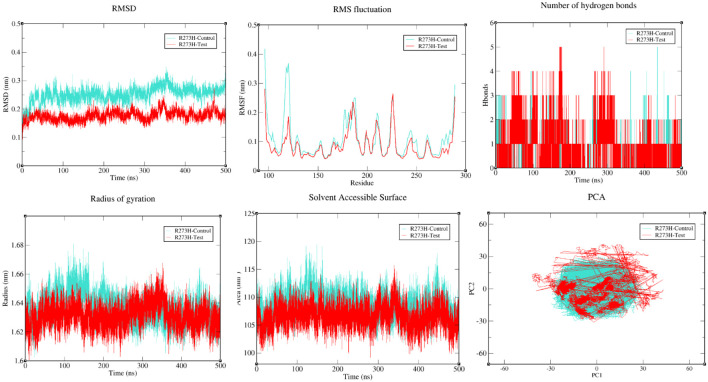
Comparative molecular dynamics analysis revealing the stabilization of the R273H contact mutant by Hydroxymycotrienin A over a 500 ns trajectory.

A comparable stabilization trend was observed in the R273H contact mutant system during the 500 ns simulation trajectory. The Hydroxymycotrienin A-bound complex exhibited substantially lower RMSD deviations and improved equilibrium behavior relative to the unbound control, which displayed extensive structural fluctuations throughout the simulation period (Figure 6A). RMSF analysis demonstrated reduced flexibility surrounding the DNA-binding interface and neighboring loop regions, indicating that ligand interaction effectively rigidified structurally dynamic segments associated with impaired DNA-contact functionality in the R273H mutant (Figure 6B). Hydrogen bond analysis revealed continuous intermolecular interaction persistence, suggesting stable ligand accommodation within the active-site cavity during the entire trajectory (Figure 6C). The ligand-bound system additionally maintained lower Rg fluctuations and reduced solvent accessibility compared with the control system, demonstrating enhanced structural compactness and reduced surface destabilization (Figures 6D,E). PCA analysis further confirmed that Hydroxymycotrienin A restricted the collective motions of the R273H mutant into a compact conformational cluster with limited phase-space exploration, whereas the control trajectory exhibited broad conformational scattering characteristic of increased dynamic instability (Figure 6F). Collectively, these simulations demonstrate that Hydroxymycotrienin A effectively suppresses mutation-associated structural perturbations and promotes conformational stabilization across both conformational and contact p53 mutants.

To quantitatively evaluate the dynamic behavior of all simulated systems, the principal molecular dynamics parameters were summarized over the 500 ns trajectories (Table 3). In the native p53 complexes, Hydroxymycotrienin A maintained structural stability comparable to APR-246, exhibiting RMSD values of 0.220 ± 0.027 nm and 0.211 ± 0.020 nm, respectively, while demonstrating substantially higher intermolecular hydrogen-bond formation (1.457 ± 0.860 *versus* 0.207 ± 0.507). In the R175H conformational mutant, Hydroxymycotrienin A reduced backbone deviations relative to APR-246, lowering the RMSD from 0.211 ± 0.044 nm to 0.181 ± 0.028 nm and decreasing RMSF values from 0.111 ± 0.080 nm to 0.097 ± 0.054 nm, indicative of reduced residue-level fluctuations. Similarly, in the R273H contact mutant, Hydroxymycotrienin A produced the lowest RMSD among all simulated systems (0.176 ± 0.017 nm), compared with 0.251 ± 0.026 nm for APR-246, while also reducing RMSF (0.086 ± 0.047 *versus* 0.107 ± 0.067 nm), decreasing SASA (106.607 ± 1.920 *versus* 108.681 ± 2.192 nm^2^), and increasing intermolecular hydrogen-bond interactions (0.722 ± 0.899 *versus* 0.364 ± 0.643). Across both mutant systems, the ligand-bound complexes maintained comparable or slightly lower radius of gyration values, suggesting preservation of structural compactness. Collectively, these quantitative analyses support the enhanced stabilizing effect of Hydroxymycotrienin A relative to APR-246, particularly in the R175H and R273H mutant p53 proteins.

**TABLE 3:** Statistical summary of molecular dynamics parameters over 500 ns simulations.

System	RMSD (nm) mean ± SD	RMSF (nm) mean ± SD	Rg (nm) mean ± SD	SASA (nm^2^) mean ± SD	H-bonds mean ± SD
Native + APR-246	0.211 ± 0.020	0.104 ± 0.057	1.638 ± 0.008	106.725 ± 1.941	0.207 ± 0.507
Native + Hydroxymycotrienin A	0.220 ± 0.027	0.108 ± 0.085	1.635 ± 0.009	107.720 ± 2.232	1.457 ± 0.860
R175H + APR-246	0.211 ± 0.044	0.111 ± 0.080	1.642 ± 0.011	107.591 ± 2.211	0.448 ± 0.696
R175H + Hydroxymycotrienin A	0.181 ± 0.028	0.097 ± 0.054	1.640 ± 0.009	107.553 ± 2.200	0.573 ± 0.841
R273H + APR-246	0.251 ± 0.026	0.107 ± 0.067	1.637 ± 0.009	108.681 ± 2.192	0.364 ± 0.643
R273H + Hydroxymycotrienin A	0.176 ± 0.017	0.086 ± 0.047	1.631 ± 0.008	106.607 ± 1.920	0.722 ± 0.899

### 3.7 Free Energy Landscape (FEL)

**FIGURE 7:**
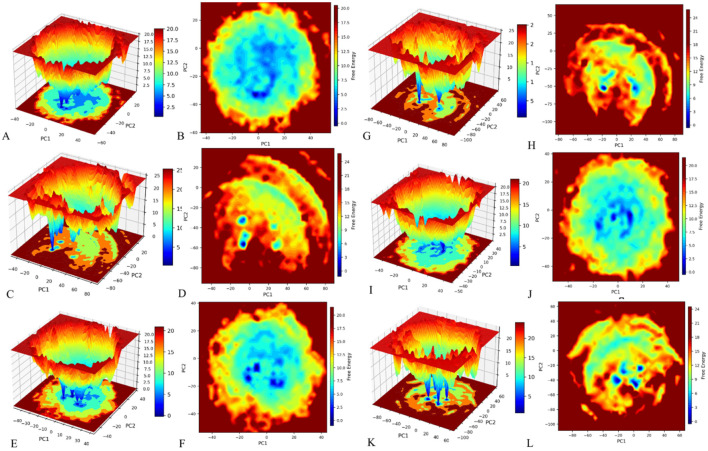
Comparative Gibbs Free Energy Landscape (FEL) plot. **(A–D)** Native p53 Complexes. **(A, B)** Native p53 bound to the reference standard APR-246; **(C, D)** Native p53 bound to the test ligand Hydroxymycotrienin **(A)**. **(E–H)** R175H Mutant Complexes: **(E, F)** R175H mutant bound to APR-246; **(G, H)** R175H mutant bound to Hydroxymycotrienin **(A)**. **(I–L)** R273H Mutant Complexes: **(I, J)** R273H mutant bound to APR-246; **(K, L)** R273H mutant bound to Hydroxymycotrienin **(A)**. Across all isoforms, the Hydroxymycotrienin A complexes **(C, D, G, H, K, L)** exhibit deeper and more confined global energy minima compared to the APR-246 complexes.

Across all six free energy landscape representations (2D and 3D) demonstrated in Figure 7, the native, 273 mutant, and 175 mutant systems, the test drug Hydroxymycotrienin A demonstrates a more favorable modulation of protein conformational dynamics compared to the control drug APR-246. Across all simulated systems, the free energy landscapes revealed distinct differences in conformational sampling between APR-246 and Hydroxymycotrienin A. The Hydroxymycotrienin A-bound complexes exhibited well-defined low-energy basins with smooth inter-basin connectivity, indicating energetically favorable conformational states. Importantly, these observations should be interpreted in conjunction with the PCA and RMSD analyses. The confined PCA distributions and reduced RMSD values observed for Hydroxymycotrienin A indicate suppression of large-scale destabilizing motions and prevention of global unfolding. At the same time, the free energy landscapes demonstrate that the protein retains access to multiple closely related low-energy conformations within a stable energetic framework. Therefore, the broader conformational space sampled by Hydroxymycotrienin A does not indicate structural instability; rather, it suggests preservation of native-like conformational plasticity that may be required for functional DNA recognition and protein activity. In contrast, APR-246-bound systems exhibited more restricted conformational sampling, reflecting a comparatively rigid energetic landscape. Collectively, these results suggest that Hydroxymycotrienin A stabilizes mutant p53 while maintaining the dynamic flexibility necessary for biological function.

## 4 Discussion

The pharmacological restoration of p53 function represents one of the most significant challenges in modern oncology, hindered historically by the protein’s elusive drug-binding topology (Bykov et al., 2017). The study addresses the challenge by identifying the *Bacillus*-derived secondary metabolite Hydroxymycotrienin A as a potential, high-affinity p53 reactivator. The most critical interpretation of the investigation is the thermodynamic superiority of this natural compound over the APR-246 parent scaffold (eprenetapopt). Molecular docking analyses revealed that Hydroxymycotrienin A achieved binding affinities of −6.63 kcal/mol (R175H) and −6.57 kcal/mol (R273H), nearly doubling the binding energy exhibited by APR-246 (∼−3.5 kcal/mol) across native and mutant isoforms. Interestingly, Hydroxymycotrienin A exhibited slightly stronger binding affinities toward the mutant proteins than the native p53 structure. This observation may be attributed to mutation-induced alterations in the local architecture of the DNA-binding domain. Structural perturbations associated with R175H and R273H can modify residue accessibility and reshape ligand-accessible cavities, thereby creating additional opportunities for favorable hydrogen-bonding, hydrophobic, and van der Waals interactions. The large and conformationally adaptable ansamycin scaffold of Hydroxymycotrienin A appears particularly well suited to exploit these mutation-induced structural features, resulting in enhanced ligand accommodation and improved binding affinity relative to the native protein. A fundamental distinction in the study lies in the mechanism of stabilization. APR-246 functions as a prodrug, converting to methylene quinuclidinone (MQ) to covalently alkylate cysteine residues (Cys124/Cys277) and lock the protein structure. This profound difference in binding energetics suggests that the complex, naturally evolved polycyclic scaffold of Hydroxymycotrienin A provides a superior geometric fit for the p53 active pocket compared to the smaller, planar, and synthetic scaffold of APR-246. The binding energies generated during virtual screening and detailed docking were not expected to be numerically identical because AutoDock Vina and AutoDock 4.2 rely on distinct scoring functions and optimization strategies (Ahmad et al., 2024). Nevertheless, both methods consistently identified Hydroxymycotrienin A as the top-performing ligand, providing confidence in the robustness of compound selection. These findings strongly support the privileged scaffold hypothesis that natural products possess an inherent evolutionary advantage in targeting complex biological macromolecules compared to synthetic combinatorial libraries (Newman and Cragg, 2020; Atanasov et al., 2021). While clinically effective, this covalent mechanism carries inherent risks. Recent clinical data suggest that APR-246 may exhibit off-target toxicity due to its indiscriminate reactivity with other cellular thiols and may encounter emerging resistance mechanisms in TP53-mutant subclones (Mishra et al., 2022). In contrast, the structural data suggest that Hydroxymycotrienin A functions as a non-covalent pharmacological chaperone. By establishing a dense, specific network of hydrogen bonds (involving Thr170 and Ser96) and Pi-alkyl interactions (with His168), it stabilizes the p53 core without the requirement for permanent chemical modification. This aligns with recent safety profiling studies advocating for a shift toward high-affinity non-covalent agents that can thermodynamically shift the folding equilibrium without the toxicity profile associated with covalent alkylator.

Mechanistically, the present findings suggest that Hydroxymycotrienin A may stabilize distinct classes of p53 mutants through mutation-specific interaction networks (Blanden et al., 2020), In the R175H conformational mutant, the ligand occupied a cavity adjacent to the L2 loop and established persistent interactions with residues including Phe212 and Val172, resulting in reduced structural fluctuations and enhanced conformational stability throughout the simulation. The observed decrease in RMSD, RMSF, and conformational dispersion suggests that the ligand functions as a molecular stabilizer capable of suppressing mutation-induced structural disorder within the DNA-binding domain (Ha et al., 2022). In contrast, the R273H contact mutant retained an overall stable fold but exhibited impaired DNA-contact functionality due to substitution of a key DNA-interacting residue. In this system, Hydroxymycotrienin A interacted with neighboring residues such as Thr211 and Asn210 and promoted a more stable DNA-binding interface while maintaining favorable conformational dynamics. These observations indicate that Hydroxymycotrienin A may exert its effects through context-dependent stabilization mechanisms, enabling structural reinforcement of conformational mutants and dynamic modulation of contact mutants through non-covalent interactions (Selivanova and Wiman, 2007).

The search for natural p53 reactivators has largely focused on plant secondary metabolites. Recent studies have identified leads such as asiatic acid, with binding energies of approximately −5.9 kcal/mol for R175H (Swargiary et al., 2023), and various curcumin analogs ranging from −5.5 to −7.0 kcal/mol (Nainggolan et al., 2025). While some phytochemicals exhibit competitive binding scores, they frequently suffer from the bioavailability gap, demonstrating high *in silico* potency but poor *in vivo* efficacy due to rapid metabolism and low gastrointestinal absorption (Farhan et al., 2023). The ADMET profiling distinguishes Hydroxymycotrienin A (ansamycin) from this group; unlike bulky polyphenols, it exhibits full compliance with Lipinski’s rule of five and is predicted to possess high metabolic stability and gastrointestinal absorption, suggesting efficacy in systemic delivery, potentially overcoming the translation hindering the advancement of many promising plant-derived leads. Moreover, Hydroxymycotrienin A demonstrated full Lipinski compliance and a high safety profile. The translational relevance of a novel p53 activator in NSCLC is heavily dictated by its ability to address Central Nervous System (CNS) metastasis, a complication affecting nearly 40% of lung cancer patients. Our ADMETlab 3.0 profiling reveals that Hydroxymycotrienin A exhibits a favorable predicted blood-brain barrier (BBB) permeability (Green signal), suggesting its potential to target brain-resident secondary tumors. Additionally, the lead demonstrated a plasma protein binding (PPB) of 73.3%, while higher than that of APR-246 (17.8%), remains within a range that ensures a sufficient free-drug fraction for therapeutic engagement. This combination of BBB penetration and acceptable PPB is a significant advantage for an ansamycin scaffold in the context of metastatic NSCLC. The reliability of these pharmacokinetic evaluations is grounded in the rigorous selection of molecular descriptors used in our computational framework. As highlighted by recent studies, comprehensive validation against diverse structural datasets is paramount for ensuring the high predictive accuracy and generalizability of ADMET models (Ahmad et al., 2025). By utilizing such a multi-validated platform, our results provide a robust theoretical baseline for the prospective development of Hydroxymycotrienin A as a systemic therapy capable of both primary tumor control and CNS protection.

Synthetic agents, such as the thiosemicarbazones NPC (NSC319726) and CTU (NSC321792), dominate the preclinical landscape. Recent computational studies report binding energies for these compounds against R175H in the range of −6.75 to −6.92 kcal/mol (Yu et al. al., 2017; Das and Mukhopadhyay, 2022). While comparable to the affinity of Hydroxymycotrienin A (−6.63 kcal/mol), these synthetic agents often function as metallochaperones or require specific intracellular redox conditions to function. Such mechanisms are inextricably linked to metal-related toxicity and oxidative stress (Xu et al., 2023). Hydroxymycotrienin A attains similar high-affinity stabilization *via* a “natural” structural compatibility, providing a means to efficacy that prevents the toxicity linked to metal-chelating synthetic pharmaceuticals.

While bacteria are prolific producers of bioactive compounds, the exploration of microbial metabolites for mutant p53 is significantly less developed than for plant or synthetic sources. Existing studies have largely focused on *Streptomyces* species, with reported binding energies ranging from −3.7 to −6.9 kcal/mol (Nivetha et al., 2022). Hydroxymycotrienin A sits at the apex of this range. Furthermore, recent reviews of the bacterial chemical space highlight that *Bacillus* species remain an underexplored reservoir for protein-protein interaction modulators (Xiao et al., 2022). Unlike marine microbial derivatives such as sulfated polysaccharides, which present significant delivery challenges due to their size and charge (Alfinaikh et al., 2025), Hydroxymycotrienin A retains a drug-like small molecule profile.

From a structural and pharmacological perspective, Hydroxymycotrienin A belongs to the triene ansamycin class of natural products, produced by *Streptomyces* and *Bacillus* species renowned for their potent anticancer and antibiotic properties (Hosokawa et al., 1996). Prototypical ansamycins, most notably Geldanamycin and its clinical derivatives, are well-characterized inhibitors of the molecular chaperone Hsp90 (Neckers, 2002). Within the context of oncological signaling, Hsp90 is known to be essential for the conformational stability of mutant p53; its inhibition typically results in the indirect destabilization and degradation of the mutant protein (Müller et al., 2005). While the known biology of the ansamycin class suggests an indirect effect on p53 *via* the Hsp90-p53 chaperone axis, our *in silico* trajectories provide compelling evidence for a novel, direct-binding mechanism to the p53 DNA-binding domain. We propose that Hydroxymycotrienin A may exhibit a multimodal therapeutic profile, potentially combining the class-characteristic Hsp90 inhibitory effect with a direct ‘molecular staple’ or chaperone activity on p53 structural mutants. This dual-targeting potential warrants further investigation to distinguish between direct p53 stabilization and indirect Hsp90-mediated effects in NSCLC models.

The ability of Hydroxymycotrienin A to effectively target both conformational (R175H) and contact (R273H) mutants represents a noteworthy pharmacological characteristic. Historically, therapeutic approaches for mutant p53 have often been developed according to mutation class, with many candidate compounds demonstrating activity predominantly against either conformational mutants or contact mutants rather than both. In contrast, the present findings suggest that Hydroxymycotrienin A maintains favorable interactions across structurally distinct mutant forms, indicating the potential for broader mutant-p53 targeting through a common non-covalent stabilization mechanism (Blanden et al., 2020). Uniquely, the docking results of the current study reveal that Hydroxymycotrienin A stabilizes the R273H mutant by engaging flanking regions without blocking the DNA-binding cleft. The study suggests a novel mode of action where the ligand acts as an allosteric clamp near the active site, restoring the functional surface potential required for DNA attraction (Balasundaram and Doss, 2022). The temporal stability observed in our 500 ns Molecular Dynamics (MD) simulations represents a significant deviation from the standard methodology found in similar literature. The vast majority of *in silico* p53 studies rely on shorter trajectories (50–100 ns), which often fail to capture protein unfolding events or ligand dissociation. As noted by Hollingsworth and Dror, extended simulations are critical for observing rare conformational transitions in intrinsically disordered regions like p53 loops (Hollingsworth and Dror, 2018). The fact that Hydroxymycotrienin A significantly dampened fluctuations in the destabilized L2 loop of the R175H mutant over this extended 500 ns duration provides a level of confidence in the complex’s stability that is largely absent in prior studies (Li et al., 2020). Additionally, Hydroxymycotrienin A acts as a pharmacological chaperone, capable of thermodynamically shifting the folding equilibrium of mutant p53 toward a functional state through robust non-covalent interactions.

Recent studies have further emphasized that extended molecular dynamics simulations combined with RMSF-based residue fluctuation analyses provide a more comprehensive assessment of protein–ligand stability than short-duration trajectories alone. Such long-timescale simulations enable the identification of persistent interaction networks, conformational adaptation, and dynamic stabilization mechanisms that may not be apparent during shorter simulations. The consistent stability observed throughout the 500 ns trajectories in the present study therefore provides additional confidence in the robustness of the predicted Hydroxymycotrienin A–p53 interactions (Ahmad and Raza, 2026).

The free energy landscape analysis revealed clear differences in the conformational behavior of the protein when bound to the test drug Hydroxymycotrienin A compared to the control drug APR-246. Hydroxymycotrienin A-bound systems consistently exhibited well-defined and energetically favorable low-energy basins with improved inter-basin connectivity, indicating stable yet dynamically adaptable conformational states. This behavior was particularly pronounced in the mutant systems, where Hydroxymycotrienin A effectively accommodated mutation-induced structural perturbations without inducing excessive energetic frustration. In contrast, APR-246 bound complexes displayed more confined conformational sampling, reflecting a comparatively rigid energy landscape (Degtjarik et al., 2021). The PCA and FEL analyses provide complementary insights into the dynamic behavior of the complexes. While PCA demonstrated that Hydroxymycotrienin A restricts large-scale destabilizing motions and prevents excessive conformational drift, the free energy landscapes revealed the presence of multiple energetically favorable conformational substates. This combination suggests that the ligand stabilizes the overall protein architecture while preserving localized conformational flexibility. Such behavior is consistent with restoration of native-like dynamic properties rather than simple rigidification of the protein structure. Consequently, Hydroxymycotrienin A appears capable of promoting both structural integrity and functional conformational adaptability within mutant p53, supporting its potential as a superior therapeutic candidate. The preservation of multiple energetically favorable conformational substates may also have important functional implications for p53 activity. DNA recognition by the p53 core domain is not a rigid process but requires a degree of conformational adaptability to accommodate sequence-specific interactions and transcriptional regulation. Therefore, the broader yet stable low-energy basins observed in the Hydroxymycotrienin A-bound complexes may represent restoration of native-like dynamic behavior that supports DNA binding while maintaining overall structural integrity. Such a balance between stability and controlled flexibility is considered essential for effective activation of downstream p53-regulated cellular defense pathways, including cell-cycle arrest, DNA repair, and apoptosis.

These findings support an important change in p53 pharmacological research. The field has long oscillated between toxic covalent modifiers (like APR-246) and potential but unstable phytochemicals. Hydroxymycotrienin A occupies the ideal middle ground: it possesses the structural complexity and specificity of a natural product with the potential of a synthetic agent. Its superior safety profile (ProTox Class 6) supports SDG 3 by offering a potential pathway towards safer, personalized oncological treatment that may improve the survival rates for patients with non-communicable diseases. One limitation of the present study is that the mutant p53 structures were generated through computational mutagenesis followed by energy minimization rather than reconstruction from experimentally resolved mutant crystal structures. Additionally, the crystallographic zinc ion was removed from all protein systems to maintain a uniform metal-free framework for comparative analysis. While this strategy enabled consistent evaluation of ligand-mediated stabilization across native and mutant p53 variants, it may not fully capture mutation-specific structural effects associated with zinc coordination and long-range conformational rearrangements. Future studies incorporating experimentally resolved mutant structures and explicit metal-ion parameterization may provide additional mechanistic insights into mutant-specific restoration processes. This research not only identifies a promising lead candidate for Non-Small Cell Lung Cancer (NSCLC) therapy but also establishes a computational framework for mining the microbial metabolome for next-generation tumor suppressor reactivators. Future investigations should prioritize wet-lab synthesis and biological validation of this compound to confirm its ability to induce downstream apoptotic targets, such as p21 and PUMA, in p53-mutant cancer models.

## 5 Conclusion

The present study successfully elucidated an integrated computational workflow to screen and validate *Bacillus*-derived secondary metabolites as modulators of the p53 tumor suppressor protein. By combining high-throughput molecular docking with extended 500 ns Molecular Dynamics (MD) simulations and Gibbs Free Energy Landscape (FEL) analysis, the study identified Hydroxymycotrienin A as a potential, dual-mechanism p53 reactivator. This evidence shows that longer time-scale simulations (500 ns) are necessary to capture the restorative dynamics of intrinsically disordered regions in mutant p53, namely, the L2 loop in the R175H mutant. In contrast, shorter trajectories were unable to fully resolve, an important methodological result of this work. The computational data provide robust evidence that Hydroxymycotrienin A thermodynamically outperforms the non-covalent structural scaffold control, APR-246. Unlike the covalent alkylating mechanism of APR-246, Hydroxymycotrienin A functions as a non-covalent pharmacological chaperone. It achieves stabilization through a “structural stapling” mechanism for the conformational R175H mutant and an “allosteric clamping” mechanism for the contact R273H mutant. Furthermore, ADMET profiling positions this compound as a Lipinski-compliant candidate with a superior predicted safety profile compared to existing synthetic modulators.

## 6 Limitation

The study provides a rational, structure-based foundation for shifting p53 drug discovery paradigms from toxic covalent modifiers toward high-affinity non-covalent binders. To definitively establish Hydroxymycotrienin A as a viable clinical candidate, future investigations must bridge the gap between these *in silico* predictions and clinical application through an explicit prospective framework for empirical validation. We formally recommend direct target engagement studies using Surface Plasmon Resonance (SPR) or Cellular Thermal Shift Assays (CETSA) to verify the proposed ‘structural staple’ or ‘allosteric clamp’ mechanism. Furthermore, the functional restoration of p53 activity must be validated in mutant human NSCLC lines through quantitative real-time PCR or Western blotting to confirm the downstream activation of canonical apoptotic targets, specifically p21 and PUMA.

Further, a significant limitation of this comparative study is the mechanistic divergence between the test lead and the reference molecule APR-246. While APR-246 functions as a prodrug that is converted intracellularly to the covalent alkylating agent methylene quinuclidinone (MQ), our analysis benchmarks Hydroxymycotrienin A against the non-covalent parent scaffold of APR-246. Consequently, our docking scores reflect the thermodynamic potential for initial non-covalent pocket recognition and structural stabilization rather than the covalent bond energy characteristic of MQ’s clinical mechanism. Hydroxymycotrienin A is proposed here as a direct-binding non-covalent pharmacological chaperone, a mechanism that avoids the requirement for metabolic activation.

## Data Availability

The original contributions presented in the study are included in the article/Supplementary Material, further inquiries can be directed to the corresponding authors.
