# Supplementary material for: Functional restoration of conformational (R175H) and contact (R273H) mutant p53 by Bacillus-derived Hydroxymycotrienin A in non-small cell lung cancer: a computational approach
Source: Front Bioinform. 2026 Jul 24;6:1875897. doi: 10.3389/fbinf.2026.1875897 (PMC13447244; doi:10.3389/fbinf.2026.1875897)
Supplement: Supplementary file 1 [file DataSheet1.pdf]

Functional Restoration of Conformational (R175H) and Contact (R273H) mutant p53 by *Bacillus*-Derived Hydroxymycotrienin A in Non-Small Cell Lung Cancer: A Computational Approach

Adeline Celina Rufus[1], Sidharth Kumar N[2], Magesh Ramasamy[2]** and Elavarashi Elangovan[1]*

[1] Medical Probiotics Lab, Department of Biotechnology, Sri Ramachandra Faculty of Biomedical Sciences & Technology, Sri Ramachandra Institute of Higher Education & Research, Chennai, Tamil Nadu, India 600116.

[2] Computational Biology Lab, Department of Biotechnology, Sri Ramachandra Faculty of Biomedical Sciences & Technology, Sri Ramachandra Institute of Higher Education & Research, Chennai, Tamil Nadu, India 600116.

*Corresponding Author: Dr. Elavarashi Elangovan, Department of Biotechnology, Sri Ramachandra Institute of Higher Education and Research (DU), Porur 600116, Chennai, Tamil Nadu, India. Email id: elavarashi@sriramachandra.edu.in

**Co-correspondence: Dr. Magesh Ramasamy, Department of Biotechnology, Sri Ramachandra Institute of Higher Education and Research (DU), Porur 600116, Chennai, Tamil Nadu, India. Email: magesh.r@sriramachandra.edu.in

Supplementary Table 1:

S.No.	Compound Name	PubChem CID	Canonical SMILES	InChlKey
1	Hydroxymycotrienin A	139587614	CC1C(C/C=C/C=C/C=CC(CC(=O)NC2=CC(=O)C=C(C2=O)CC/C=C\C1O)OC)OC(=O)[C@@H](C)NC(=O)C3CCCCC3	TZUOLVRFZJPGRC-CJUNPOOESA-N
2	(1R,2R,4aS,4bR,6aS,10aS,12aS)-2,4b,7,7,10a,12a-hexamethyl-1-[3-(4-methylphenyl)butyl]-3,4,4a,5,6,6a,8,9,10,10b,11,12-dodecahydro-1H-chrysen-2-ol	139583555	CC1=CC=C(C=C1)C(C)CC[C@@H]2[C@]3(CCC4[C@]([C@@H]3CC[C@@]2(C)O)(CC[C@@H]5[C@@]4(CCCC5(C)C)C)C)C	UIYOFGICAOMDQH-LVXDOFCUSA-N
3	Macrolactin I	6390938	CC1CCC/C=C/C=C/C(CC(C/C=C/C=C/C(C/C=C/C=C\C(=O)O1)O)O)O	XXDIJWSZFWZBRM-BPFVYSANSA-N
4	Onoceranoxide	21672657	C[C@]12CCCC([C@@H]1CC[C@@]3([C@@H]2CC[C@@H]4[C@]5(CCCC([C@@H]5CC[C@]4(O3)C)(C)C)C)C)(C)C	WKJQOTFJIQYADY-QPYQYMOUSA-N
5	Cereusitin A	132494419	CC(C)C[C@H]1C(=O)N2C[C@@H](C[C@H]2C(=O)N[C@H](C(=O)N3C[C@@H](C[C@H]3C(=O)N1)O)CC4=CC=CC=C4)	IIYSUNCOHRYFBU-OFELHODLSA-N

			O	
6	Sporulene B	102596711	CC1=CC=C(C=C1)[C@H](C)CC[C@H]2C(=C)CC[C@@H]3[C@@]2(CC[C@H]4[C@]3(CC[C@@H]5[C@@]4(CCCC5(C)C)C)C)C	ZRZKSUXNULZPHV-ATJBQKKCSA-N
7	Hydroxymycotrienin B	139583958	CC1C(C/C=C/C=C/C=CC(CC(=O)NC2=CC(=O)C(=O)C(=C2O)CC/C=C(\\C1OC(=O)C(C)NC(=O)C3CCCCC3)/C)OC)O	GGZCXNDCJRHBKJ-IGFODWNLSA-N
